# Burden of cytomegalovirus reactivation post kidney transplant with antithymocyte globulin use in Thailand: A retrospective cohort study

**DOI:** 10.12688/f1000research.16321.1

**Published:** 2018-09-28

**Authors:** Maria N. Chitasombat, Siriorn P. Watcharananan

**Affiliations:** 1 Division of Infectious disease, Department of Medicine, Faculty of Medicine, Ramathibodi Hospital, Mahidol University, Bangkok, 10400, Thailand

**Keywords:** antithymocyte globulin, cytomegalovirus, burden, kidney transplantation

## Abstract

**Background:** Cytomegalovirus (CMV) is an important cause of infectious complications after kidney transplantation (KT), especially among patients receiving antithymocyte globulin (ATG). CMV infection can result in organ dysfunction and indirect effects such as graft rejection, graft failure, and opportunistic infections **.** Prevention of CMV reactivation includes pre-emptive or prophylactic approaches. Access to valganciclovir prophylaxis is limited by high cost. Our objective is to determine the burden and cost of treatment for CMV reactivation/disease among KT recipients who received ATG in Thailand since its first use in our center.

**Methods: **We conducted a single-center retrospective cohort study of KT patients who received ATG during 2010-2013. We reviewed patients’ characteristics, type of CMV prophylaxis, incidence of CMV reactivation, and outcome (co-infections, graft function and death). We compared the treatment cost between patients with and without CMV reactivation.

**Results:** Thirty patients included in the study had CMV serostatus D+/R+. Twenty-nine patients received intravenous ganciclovir early after KT as inpatients. Only three received outpatient valganciclovir prophylaxis. Incidence of CMV reactivation was 43%, with a median onset of 91 (range 23-1007) days after KT. Three patients had CMV end-organ disease; enterocolitis or retinitis. Infectious complication rate among ATG-treated KT patients was up to 83%, with a trend toward a higher rate among those with CMV reactivation ( *P* = 0.087). Patients with CMV reactivation/disease required longer duration of hospitalization ( *P* = 0.018). The rate of graft loss was 17%. The survival rate was 97%. The cost of treatment among patients with CMV reactivation was significantly higher for both inpatient setting ( *P* = 0.021) and total cost ( *P* = 0.035) than in those without CMV reactivation.

**Conclusions:** Burden of infectious complications among ATG-treated KT patients was high. CMV reactivation is common and associated with longer duration of hospitalization and higher cost.

## Introduction

Human cytomegalovirus (CMV) is an important cause of infectious complications after kidney transplantation (KT) ^1^. CMV infection can result in end-organ diseases and indirect effects such as opportunistic infections, graft rejection, and graft failure ^2^. Since the introduction of antithymocyte globulin (ATG) in transplantation, the incidence of CMV reactivation has increased up to 10–50% ^3– 9^. To prevent CMV reactivation, prophylactic and pre-emptive approaches are almost equally effective. CMV prophylaxis reduces the incidence of CMV disease and associated mortality in solid organ transplant recipients ^10^. However, the cost of prophylaxis is high. Data from our center prior to ATG use showed that the incidence of symptomatic CMV reactivation among KT recipients (CMV D+/R+), was low (4.6%) ^11^. In recent years, the use of ATG has been implemented nationwide.

In this study performed in Thailand, we evaluated the burden of symptomatic CMV reactivation/disease following the use of ATG in situations where CMV prophylaxis was not widely available and affordable. We also evaluated the outcome of ATG-treated patients in terms of infectious complications, graft loss, and cost of treatment in patients who developed CMV reactivation/disease and those who did not.

## Methods

This was a retrospective cohort study of all ATG-treated (induction/antirejection therapy) KT patients aged ≥15 years at Ramathibodi Hospital, Bangkok, Thailand between January 2010 and July 2013. At our institution, routine oral antimicrobial prophylaxis included 1 year acyclovir (withheld during the period of anti-CMV exposure), 9 months isoniazid and 1 year cotrimoxazole. The strategy for CMV prophylaxis or pre-emptive therapy was based on the physician’s decision. Blood CMV viral load was monitored.

This study was approved by the Institutional Ethics Committee of Ramathibodi Hospital, Mahidol University (#12-56-24). For this retrospective study, formal informed consent was not required by the committee.

### Data collection

We collected data from the records of patients on: demographic characteristics; underlying disease; type of KT; details of induction regimen and maintenance immunosuppression; serum creatinine; CMV serostatus of donors and recipients; CMV prophylaxis; clinical course; post-KT infectious complications; graft rejection; laboratory parameters at the time of CMV reactivation/disease (complete blood count, chemistry, liver function tests, immunosuppressive drug level, plasma CMV viral load) (COBAS Amplicor Monitor test; Roche Molecular Diagnostics); and treatment for CMV reactivation/disease. Outcomes including infectious complications, graft rejection, and death were measured at 3 and 6 months after KT until the end of the study in January 2014.

The cost of transplantation was analyzed among 26 KT patients (excluding four with missing data). We collected data for ganciclovir/valganciclovir use (duration and dosage) and medical expenses (overall cost of hospitalization and treatment, outpatient visits, emergency room visits, medication, laboratory tests, and imaging) from the initial hospital admission for KT until 6 months and at the end of study in January 2014. The direct cost of treatment for CMV infection/disease was not available because there was no system in the hospital to extract the specific data. We calculated the cost in US$ (2014 conversion rate of 32.506 THB to 1 US$) of prophylaxis with valganciclovir, with dose adjustment for glomerular filtration rate (GFR) for each patient according to serum creatinine at discharge.

### Definition

Definition of CMV reactivation/disease was based on that of Ljungman *et al.* ^2^. CMV reactivation was defined as new detection of CMV infection (plasma CMV viral load was used in this study) in patients who had previously had CMV serostatus positive (R+). CMV gastrointestinal disease was defined by combination of gastrointestinal symptoms, endoscopic mucosal lesions, and demonstration of CMV infection by histopathological examination, immunohistochemical analysis, or *in situ* hybridization of gastrointestinal tract biopsy specimens. CMV retinitis was diagnosed by an ophthalmologist from examination of typical lesions.

### Statistics

Data were presented as median (range) and number (%). Categorical variables among patient groups were compared using the χ ^2^ or Fisher’s exact test, and continuous variables were compared using the Mann–Whitney U test. Statistical analyses were performed by SPSS software version 17.0 (IBM SPSS Statistics, Chicago, Illinois, USA).

## Results

A total of 30 KT patients received ATG during the study period. Patients’ characteristics are shown in Table 1. The majority of patients (n = 26; 87%) resided in rural areas. Six (20%) had a second KT, and 16 (53%) had living donor KT. ATG was used for induction therapy in 23 (77%) patients and antirejection therapy in seven. The total median ATG dose was 225 (105-700) mg. The maintenance regimen included mycophenolate mofetil, tacrolimus and prednisolone (n = 22, 73.3%); mycophenolate mofetil, cyclosporine and prednisolone (n = 4, 13.3%); cyclosporine, everolimus and prednisolone (n = 2, 6.6%); sirolimus, mycophenolate mofetil and prednisolone (n = 1, 3.3%); and everolimus, mycophenolate mofetil and prednisolone (n = 1, 3.3%). Delayed graft function occurred in 13 (43.3%) patients. Inpatient post-KT CMV prophylaxis with intravenous ganciclovir was given to 29 (96.6%) patients for a median duration of 13 (2-55) days. The median duration of hospitalization post-KT was 28 (16-78) days. Upon discharge, 16 (53%) patients had impaired graft function [GFR 40-59 ml/min in six (20%) patients, and 25-39 ml/min in five (17%) and 10-24 ml/min in five]. Two patients required hemodialysis at discharge because of early graft loss from severe antibody-mediated rejection. Outpatient CMV prophylaxis with valganciclovir was given to three (10%) patients. Rejection was diagnosed in 13 (43%) patients, but only 10 (76.9%) cases were confirmed by kidney biopsy. The median time to diagnosis of rejection was 13 (1-266) days. The types of rejection in 10 patients included antibody-mediated rejection (80%), cellular rejection (10%), and combined antibody and cellular rejection (10%). The details of antirejection therapy are described in Table 1.

**Table 1.** Patients’ baseline characteristics, treatment and outcome (n = 30).

Parameters	N (%)
Age (median, range; years)	51 (25–68)
Gender, male	13 (43)
**Cause of end-stage renal disease**	
Idiopathic	16 (53)
Glomerulonephritis	9 (30)
Diabetic nephropathy	3 (10)
Others ^†^	2 (7)
CMV D+/R+	30 (100)
**Immunologic risks**	
ABO incompatibility	1 (3)
HLA mismatches >3	13 (43)
PRA >10%	15 (50)
Cold ischemic time (median, range; minutes)	39 (7–8640)
**Induction therapy (n = 29)**	
Anti-thymocyte globulin	23 (77)
IL-2 antagonist	5 (17)
Others ^‡^	
**Anti-rejection therapy (n=13)**	
Pulse methylprednisolone	7 (54)
Anti-thymocyte globulin	7 (54)
IVIG	5 (38)
Plasmapheresis	6 (46)
Adjustment of drug dosages/level ^§^	4 (30)
Combination of the regimen ^||^	6 (46)
Cold ischemic time (median, range; minutes)	39 (7–8640)
**Immunosuppressive agents**	(dose, mg/day)
Cyclosporine (n=5, 30%)	150 (95–275)
Mycophenolate (n=27, 90%)	1500 (1000–2000)
Tacrolimus (n=21, 70%)	5 (1.5–9)
Prednisolone (n=29, 97%)	20 (5–40)
Others ^§^ (n=2, 6%)	
Serum Cr at KT discharge (median, range; mg/dL), (n=28)	1.56 (0.39–5.89)
**Outcome**	
Serum creatinine at follow-up (median, range;mg/dL)	1.37 (0.65–6.95)
Duration of follow-up after KT (median, range; days)	542 (134–1583)
Graft loss (n, %)	5 (16)
Time to graft loss (median, range; days)	266 (41–1038)
Death (n, %)	1 (0.03)

^†^Renal calculi and polycystic kidney disease; ^‡^ATG and IL-2 antagonist (n=1), IL-2 antagonist, rituximab and bortezemib (n=1); ^§^sirolimus (n = 1, 3%; 1 mg/day), everolimus (n = 3, 10%; 3 (2–4) mg/day); ^||^pulse methylprednisolone and ATG (1, 8%), pulse methylprednisolone, ATG, IVIG and plasmapheresis (3, 23%), ATG and plasmapheresis (1, 8%), ATG, IVIG and plasmapheresis (1, 8%). D+, Donor CMV seropositive; R+ recipient CMV seropositive; HLA, human leukocyte antigen; PRA, panel reactive antibody; IVIG, Intravenous immunoglobulin; Cr, creatinine; KT, kidney transplantation.

The median duration of follow-up was 542 (134-1583) days after KT. None of the patients who received valganciclovir prophylaxis developed CMV reactivation/disease. Thirteen patients developed CMV reactivation/disease. Six (46%) had low-grade CMV viremia without end-organ disease that spontaneously resolved after reduced immunosuppression. Four patients had CMV viremia plus fever, leukopenia and thrombocytopenia, which were compatible with CMV syndrome. Three patients had CMV end-organ disease: two with gastrointestinal disease and one with retinitis. The median onset of CMV reactivation/disease was 91 (23-1007) days after KT. Seven patients required anti-CMV therapy with a median duration of 25 (2-75) days, and intravenous ganciclovir for 12 (2-56) days. Laboratory parameters at the time of CMV reactivation/disease were: median white blood cell count 6,585 (3,082-9,962) cells/mm ^3^; 11 patients (85%) had lymphopenia, with a median absolute lymphocyte count of 519 (322-1,252) cells/mm ^3^; and median serum creatinine was 1.37 (0.65–6.95) mg/dl. Infectious complications occurred in 25 (83%) patients ( Table 2). *Pneumocystis jirovecii* pneumonia occurred in four patients who did not received cotrimoxazole at the time of diagnosis. Only one patient (ABO incompatibility) died at 266 days after KT because of several infectious complications ( *Pseudomonas aeruginosa* septicemia, *P. jirovecii* pneumonia, invasive pulmonary aspergillosis, and disseminated *Mycobacterium abscessus* infection). Patient outcomes are shown in Table 1. ATG-treated KT patients with CMV reactivation/disease required longer duration of hospitalization after KT, with a median duration of 40 (21–78) days compared with patients without CMV reactivation of 26 (16–61) days ( *P* = 0.018).

**Table 2.** Complications among ATG-treated KT recipients (n = 30).

Characteristics	No CMV (n =17)	CMV (n=13)	*P*-value
**Infectious complications** **(no, %)**	14 (82)	11 (85)	0.087
Bacterial	12 (71)	8 (62)	0.705
Fungal ^¶^	3 (18)	4 (31)	0.666
Non-CMV viruses ^†^	2 (12)	2 (15)	>0.99
Mycobacterium ^‡^	1 (6)	2 (15)	0.565
PJP	3 (18)	1 (8)	0.613
**Rejection (no, %)**	7 (41)	6 (46)	0.785
Timing of rejection (median; range, days)	41 (2–266)	13 (1–249)	0.668
**Graft loss (no, %)**	4 (24)	1 (8)	0.355
Time to graft loss (median; range, days)	332 (41–1038)	2081 (208–2051)	0.48

No CMV, patients with no evidence of CMV reactivation/diseases; CMV, patients with CMV reactivation/diseases. ^¶^ *Candida* urinary tract infection (n = 3), candidemia (n = 2), invasive pulmonary aspergillosis (n=1), and disseminated histoplasmosis (n = 1). ^†^BK-virus-associated nephropathy (n = 1), parvovirus-B19-associated pure red cell aplasia (n = 1), disseminated varicella zoster infection (n = 1), and rhinovirus lower respiratory tract infection (n = 1). ^‡^Disseminated *Mycobacterium tuberculosis* infection (n = 1), *Mycobacterium hemophilum* soft tissue infection (n = 1), disseminated *Mycobacterium abscessus* infection (n = 1) PJP, *Pneumocystis jirovecii* pneumonia

The cost of KT was analyzed among 26 patients (excluding four with missing data) ( Table 3). The cost of 100-day inpatient post KT, total inpatient post KT, and total post KT was significantly higher among patients with CMV reactivation/disease ( *P* < 0.05). The cost of valganciclovir for patients with normal GFR (900 mg/day) for 100 and 180 days was US$ 7,900 and US$ 14,220, respectively. We calculated the median cost of valganciclovir prophylaxis according to GFR in each patient upon discharge of KT to 100-day and 200-day was US$2716 (range; US $210-6,336), and US $5,431 (range; US $420-12,673), respectively.

**Table 3.** Cost-outcome of ATG-treated KT recipients with/without CMV reactivation/diseases (n=26).

Costs (in US$)	All recipients (n=26)	CMV (n=12)	No CMV (n=14)	*P-*value
100-day post KT	Median (range)	Median (range)	Median (range)	
Inpatient	18,667 (7,629–77,314)	22,088 (13,731–77,314)	15,565 (7,629–43,716)	0.027
Outpatient	3,928 (689–11,029)	3,671 (1,763–5,208)	4,637 (689–11,029)	0.382
Sum	21,390 (12,345–79,078)	25,174 (17,389–79,078)	19,871 (12,345–44,405)	0.100
180-day post KT				
Inpatient	19,923 (8,553–77,300)	23,288 (14,613–77,300)	17,460 (8,553–43,716)	0.051
Outpatient	7,432 (766–22,259)	7,213 (4,364–22,259)	7,783 (766–18,001)	0.837
Sum	25,530 (15,811–81,664)	29,426 (21,479–81,664)	24,159 (15,965–44,482)	0.100
Total post KT				
Inpatient	21,071 (8,553–77,300)	24,847 (14,612–77,300)	18,796 (8,553–43,887)	0.021
Outpatient	16,894 (1,093–42,533)	18,031 (4,364–42,533)	16,150 (1,093–32,253)	0.681
Sum	39,791 (23,049–116,780)	42,712 (25,737–116,780)	34,614 (23,049–55,137)	0.035

KT, kidney transplantation; *P* value calculated by Mann–Whitney U test.

Raw data for the study ‘Burden of cytomegalovirus reactivation post kidney transplant with antithymocyte globulin use in Thailand: A retrospective cohort study Click here for additional data file. Copyright: © 2018 Chitasombat MN and Watcharananan SP 2018 Data associated with the article are available under the terms of the Creative Commons Zero "No rights reserved" data waiver (CC0 1.0 Public domain dedication).

## Discussion

There is a lack of data about the burden of CMV reactivation/disease among KT recipients with CMV D+/R+ treated with ATG in Thailand. In Thailand, CMV prophylaxis is not widely available because of the high cost of valganciclovir. Pre-emptive treatment with plasma CMV viral load monitoring is difficult to achieve because of the need for frequent visits to the transplantation center. Our study is believed to be the first in Thailand to assess the burden of CMV reactivation after KT with ATG treatment. The incidence of CMV reactivation among CMV D+/R+ patients in our study was higher than in a previous study from our center prior to the use of ATG (53% vs.16.5 %) ^11^. The incidence was similar to that in studies from Kuwait (43%) and Germany (53.8%) ^12, 13^. CMV reactivation is known to have immunomodulatory effects in transplant patients, resulting in allograft dysfunction and other infectious complications ^2^. The overall rate of opportunistic infection is high in ATG-treated patients, and there is a trend toward higher co-infection rates among patients with CMV reactivation/disease. The rate of graft rejection/loss was not increased among patients with CMV reactivation. However, the lack of statistical power that resulted from the small number of patients makes it impossible to draw firm conclusions.

Financial burden is a major problem in resource-limited settings. We performed a preliminary analysis of outcome in terms of cost, differentiating among patients with and without CMV reactivation/disease. We could not perform a full health economic analysis because of the small sample size. We demonstrated that the cost of KT was higher among patients with CMV reactivation/disease as a result of longer hospitalization, which was partly related to treatment of infectious complications. The cost within 100 days after KT was high (US$ 18,667) compared with that in a study from Chile (US $11,186) ^14^. The possible reasons were the use of high-cost treatment including ATG, intravenous immunoglobulin, rituximab, and plasmapheresis in our study. A study from the US with similar patients showed that the cost was high, up to US$ 49,000 per admission ^15^. In a study from Australia, the cost at 12 months after KT was AU$ 89,188, 85,227, and 88,860 for no induction, induction with anti-interleukin-2, and induction with ATG, respectively ^16^ *.* Most studies have focused on the cost of induction/rejection therapy; however, the cost after KT including treatment of infectious complications was not included.

One analysis has shown that universal CMV prophylaxis is cost-effective in KT patients with CMV R+ ^17^. The lack of CMV prophylaxis could lead to even higher costs because of the cost of hospitalization among CMV D+/R– patients ^18^. A study from China revealed that the cost of KT was mainly related to drug treatment rather than hospitalization, which is different from the situation in western countries ^19^. In our study, the lack of universal valganciclovir prophylaxis in KT patients who received ATG induction was because of the high cost of medication. The alternative pre-emptive approach with monitoring CMV viral load for R+ patients has been described as an effective strategy ^18^. However, in our center an adequate pre-emptive approach was not possible because of the need for frequent follow-up visits, which were not feasible for most patients who resided in rural areas.

An economic model that simulated long-term costs and outcomes of prolonged prophylaxis with valganciclovir in a cohort of 10,000 D+/R– KT patients revealed that 200-days prophylaxis was more cost-effective than a 100-day regimen, with drug cost estimated based on normal GFR ^20^. In our study, most patients had impaired graft function (low GFR) as a result of the extended use of deceased donors ^21^. We calculated that the cost of valganciclovir prophylaxis according to low GFR at discharge up to 100-day was substantially less than the cost estimated with normal GFR. Our study demonstrated that the cost after KT among patients with CMV reactivation/disease was significantly higher than in those without CMV reactivation/disease.

The limitations of our study included a small sample size from a single-center retrospective study, and cost or sensitivity analysis of the cost-effectiveness was not intended. The heterogeneity of our patients was high, which limited the economic conclusions of this study. Another potential bias was the use of rituximab in few patients and the duration of follow-up time after KT. Generalization of our data should be done with caution, depending on the institutional protocol for KT. Future CMV prophylaxis or pre-emptive treatment in resource-limited settings should be evaluated in a prospective study with a larger sample size.

## Conclusions

Our study highlighted the burden of CMV reactivation/disease and opportunistic infections in ATG-treated KT patients in a developing country where routine CMV prophylaxis may not be affordable.

## Data availability

The data referenced by this article are under copyright with the following copyright statement: Copyright: © 2018 Chitasombat MN and Watcharananan SP

Data associated with the article are available under the terms of the Creative Commons Zero "No rights reserved" data waiver (CC0 1.0 Public domain dedication). 

Dataset 1: Raw data for the study ‘Burden of cytomegalovirus reactivation post kidney transplant with antithymocyte globulin use in Thailand: A retrospective cohort study’, 10.5256/f1000research.16321.d219028 ^22^
