# Supplementary material for: Burden of cytomegalovirus reactivation post kidney transplant with antithymocyte globulin use in Thailand: A retrospective cohort study
Source: F1000Res. 2018 Sep 28;7:1568. [Version 1] doi: 10.12688/f1000research.16321.1 (PMC6234719; doi:10.12688/f1000research.16321.1)
Supplement: Peer review history

# Peer review: Referee response for version 1

**DOI:** 10.5256/f1000research.17829.r38839

**Published:** 2018-11-12

**Authors:** Adisorn Lumpaopong

**Affiliations:** 1 Department of Pediatrics, Phramongkutklao Hospital, Bangkok, Thailand

This study mentions about burden of CMV reactivation in KT patients who underwent transplantation. All patients had serology as D+/R+ and received ATG as induction. Since ATG is a highly immunosuppressive agent, the results of this study reveals CMV reactivation is common and other infectious complications also high. Medical expenses demonstrates different among CMV reactivation and without CMV group. As we know, cost of oral valganciclovir is expensive and need long period for prophylaxis. Preemptive treatment and frequent follow up might provide benefits in this patient group in Thailand that will provide cost saving for this situation.

I have read this submission. I believe that I have an appropriate level of expertise to confirm that it is of an acceptable scientific standard.

# Peer review: Referee response for version 1

**DOI:** 10.5256/f1000research.17829.r38840

**Published:** 2018-10-03

**Authors:** Siraya Jaijakul

**Affiliations:** 1 Department of Medicine, Houston Methodist Hospital, Houston, TX, USA

In this manuscript Chitasombat and Watcharananan conducted a study of the burden and cost of treatment for CMV reactivation/disease among CMV serostatus D+/R+ kidney transplant (KT) recipients who received antithymocyte globulin (ATG) in a single-center in Thailand, which has limited healthcare resources. The study has showed that due to high cost of valganciclovir, patients were not able to afford the medication for using as universal CMV prophylaxis post KT. Consequently, since ATG could increase risk of developing CMV reactivation/disease in KT recipients, patients required longer hospitalization resulting in more financial burden.  

 The manuscript is well-written and well-organized.

 As mentioned by authors, generalization of the study is limited by a small sample size from a single-center setting. The result of 180-day inpatient cost post KT was not significant higher in CMV reactivation/disease group which could be due to a small sample size but we still could see the trend of higher financial burden among CMV reactivation/disease group.  

 A prospective study with a larger sample size or even in multi-center setting is warranted since this could impact treatment approaches for KT patients in Thailand. If CMV prophylaxis or pre-emptive treatment could be done, it would be interesting to see what would be an outcome and how much would it be for the cost of a serial CMV PCR monitoring and outpatient visits in the setting.

I have read this submission. I believe that I have an appropriate level of expertise to confirm that it is of an acceptable scientific standard.
